# Behavioral contagion in social games: how others’ attitude modulate our actions

**DOI:** 10.3389/fpsyg.2026.1790974

**Published:** 2026-03-10

**Authors:** Cecilia Carapelli, Francesca Tambuscio, Bernardo Rossi, Alessio Saccuti, Giuseppe Di Cesare

**Affiliations:** 1 Department of Food and Drugs, University of Parma, Parma, Italy; 2 Department of Medicine and Surgery, University of Parma, Parma, Italy; 3 Italian Institute of Technology, Cognitive Architecture for Collaborative Technologies Unit, Genova, Italy

**Keywords:** affective neuroscience, games, interaction mechanism, motor performance, social perception

## Abstract

Human social interaction relies fundamentally on the ability to express and perceive affective states through action. According to Daniel Stern’s concept of Vitality Forms (VFs), actions convey affective qualities, such as gentleness or rudeness, shaping how people behave beyond their action goal. While previous research has shown that VFs expressed by agents can influence observers’ perception and their motor responses, evidence from real, face-to-face social interactions remains limited. The present study addresses this gap by examining how affective contagion mediated by VFs operates during an authentic social exchange. Specifically, this work aims to: 1) identify the kinematic parameters that are influenced by others during a real game interaction; 2) determine whether this contagion effect is conscious or automatic; 3) examine whether this effect is associated with participants’ empathy abilities. In this view, pairs of participants engaged in a tic-tac-toe game, with one player acting as an insider instructed to consistently display either gentle or rude behavior through their action execution. Across 18 matches, the action of both players were recorded to assess whether and how the VFs of the inside player modulated the participant’s motor response in terms of kinematics. At regular intervals, participants reported their own affective state and their perception of the insider’s affect using Likert scales. Moreover, to assess individual empathic abilities, the Empathy Quotient questionnaire was administered. The findings revealed three principal results. First, participants’ actions were significantly modulated by the VFs of the inside player across multiple kinematic parameters (i.e., velocity, acceleration etc.), indicating a clear motor contagion effect. Second, although participants accurately perceived changes in the insider’s attitude, they did not report corresponding changes in their affective state, suggesting that VFs contagion occurs implicitly and without conscious awareness. Third, the magnitude of this motor contagion was positively correlated with empathic abilities of participants.

## Introduction

Regarding social interaction, a fundamental aspect is the ability to communicate our intentions and affective states, as well as to interpret those of others, through our actions. Stern (1985, 2010) used the term “*Vitality Forms”* (VFs) to describe this affective component of actions, which play a dual role in social interaction: enabling agents to express their internal states and enabling observers to understand the internal states of others. This affective nuance of action has been conceptualized as the *how* component, capturing qualitative aspects of action that go beyond its goal (*what*) and intention (*why*). For example, a simple gesture such as a handshake can be performed in a weak or vigorous manner, each conveying a distinct affective message. These different aspects of the action not only can be distinct conceptually, but also at neural level. In this regard, Di Cesare et al. (2014) demonstrated that, during the observation of actions, paying attention to the goal activated regions of the parieto-frontal mirror regions (Rizzolatti et al., 2014), whereas focusing on the action VFs induced the activation of the dorso-central insula (DCI) (Di Cesare et al., 2014). Moreover, the same authors conducted other fMRI studies demonstrating that this region is activated not only for the observation but also for the execution of actions conveying different VFs (Di Cesare et al., 2015, 2021, 2024). To date, new studies have shown that DCI may be part of a more extended pathway involved in the encoding of VFs actions such as the dorsolateral prefrontal cortex (PFC) and the premotor cortex (PM) (Di Cesare et al., 2024, 2025). Due to the importance of VFs in social life, in the last years, studies have begun to investigate whether and how VFs expressed by an agent influence the motor behavior of the receiver. Previous researchers suggest that action understanding relies on a motor simulation (Fadiga et al., 1995; Gallese et al., 1996; Rizzolatti et al., 2014), where observing an action triggers an internal copy that helps to predict its goal. This hypothesis indicates that when the motor system responds in a social context, the resulting action may automatically reflect the characteristics of the observed movement, producing a motor contagion effect (Chartrand and Bargh, 1999; Iacoboni, 2009; Bisio et al., 2014). From this perspective, it is plausible to hypothesize that observing gentle or rude action VFs may modulate the kinematic features of the observer’s motor response accordingly. In this respect, Di Cesare et al. (2017) conducted a kinematic study in which participants received a gentle or rude requests (“take it” or “give me”) and were required to produce an action response taking or giving a bottle placed in front of them. The results showed that the gentle or rude requests significantly influenced action execution. Specifically, following a rude request, participants interacted with the object with higher movement velocity and a larger trajectory, whereas following a gentle request they produced a softer interaction, characterized by lower velocity and a smaller trajectory. However, it is important to note that human interactions involve not only motor aspects but also perceptual ones, which are equally important. Consequently, Lombardi et al. (2021) conducted another study to examine whether, beyond action execution, VFs expressed by the agent also influence the receiver’s perception. The participants were instructed to perform two tasks: in the first one they received rude or gentle requests made through physical contact or vocally and then they had to estimate the end of a passing action observed on a monitor; in the second task they had to pass an object in response to a video request. Results of this study clearly demonstrate that VFs expressed by an agent affect not only the motor behavior of the receiver but also their perception.

Nevertheless, evidence of this contagion effect comes from exclusively virtual or pseudo-social interaction performed in highly controlled experimental settings. To date, it remains unexplored how affective contagion driven by VFs unfolds during real, face to face social interactions. Specifically, this work aims to investigate three fundamental key points: 1) assess how kinematic features are affected by others during a real interaction; 2) establish whether the VFs contagion effect is conscious or automatic; 3) understand if this effect is associated with participants’ empathy abilities. For this purpose, we carried out a kinematic experiment in which participants played tic-tac-toe in pairs. Each pair consisted of one participant and one experimenter acting as a player (inside player), who had been instructed to modulate his behavior in a rude or gentle manner during the various matches. The study comprised 18 matches, 9 played with a gentle attitude and 9 played with a rude attitude by the inside player. During the game, the action kinematics of both players were recorded. After every three matches, participants evaluated their own internal state and that of the other (inside player) using two Likert scales. Our main hypothesis was that the VFs expressed by the inside player through his game’s actions would significantly modulate participants’ action kinematics such as velocity and acceleration, both positively and negatively. It will be important to determine whether this potential contagion, driven by the VFs performed by the inside player, is a conscious or automatic process and related to the empathic abilities of participants.

## Methods

### Participants

The present study was performed on 18 healthy right-handed volunteers (10 females; mean age = 23.2 years; SD = ±1.6 years). The participants were mainly students, native Italian speakers and had normal or corrected-to-normal vision and normal hearing. No neurological or cognitive disorders were reported, and none of the participants were undergoing psychopharmacological treatment. Moreover, all participants were also tested for their empathic abilities by using the Empathic Quotient Scale-Short Version (EQ-Short) (Wakabayashi et al., 2006; Italian version Senese et al., 2016) and one participant was excluded from the study due to extreme outlier values (EQ total score < 3SD). The study received approval by the ethical committee of University of Parma (Prot.145816, 52-2024-N) and was carried out according to the principles expressed in the Declaration of Helsinki. The participants provided written informed consent.

### Psychological test

To assess individual empathy level and examine a possible correlation with the game performance, participants completed some psychological questionnaires before starting the experiment. Most importantly, the EQ-Short scale was administered to participants beforehand through a brief online form. This scale is a reduced version of the original Empathy Quotient developed by Baron-Cohen (Lawrence et al., 2004). The EQ scale assessed empathy as a stable personality trait using 15 items. Participants rated their level of agreement with each statement on a 4-point Likert scale (0 = strongly disagree to 3 = strongly agree). For example, one item reads: ‘I quickly and intuitively tune in to what others are feeling.” The scale comprises three subscales, cognitive empathy (CE), emotional responsiveness (ER), and social skills (SS). Total scores range from 0 to 45, with higher scores indicating greater empathy.

### Experimental setting and paradigm

**Figure 1:**
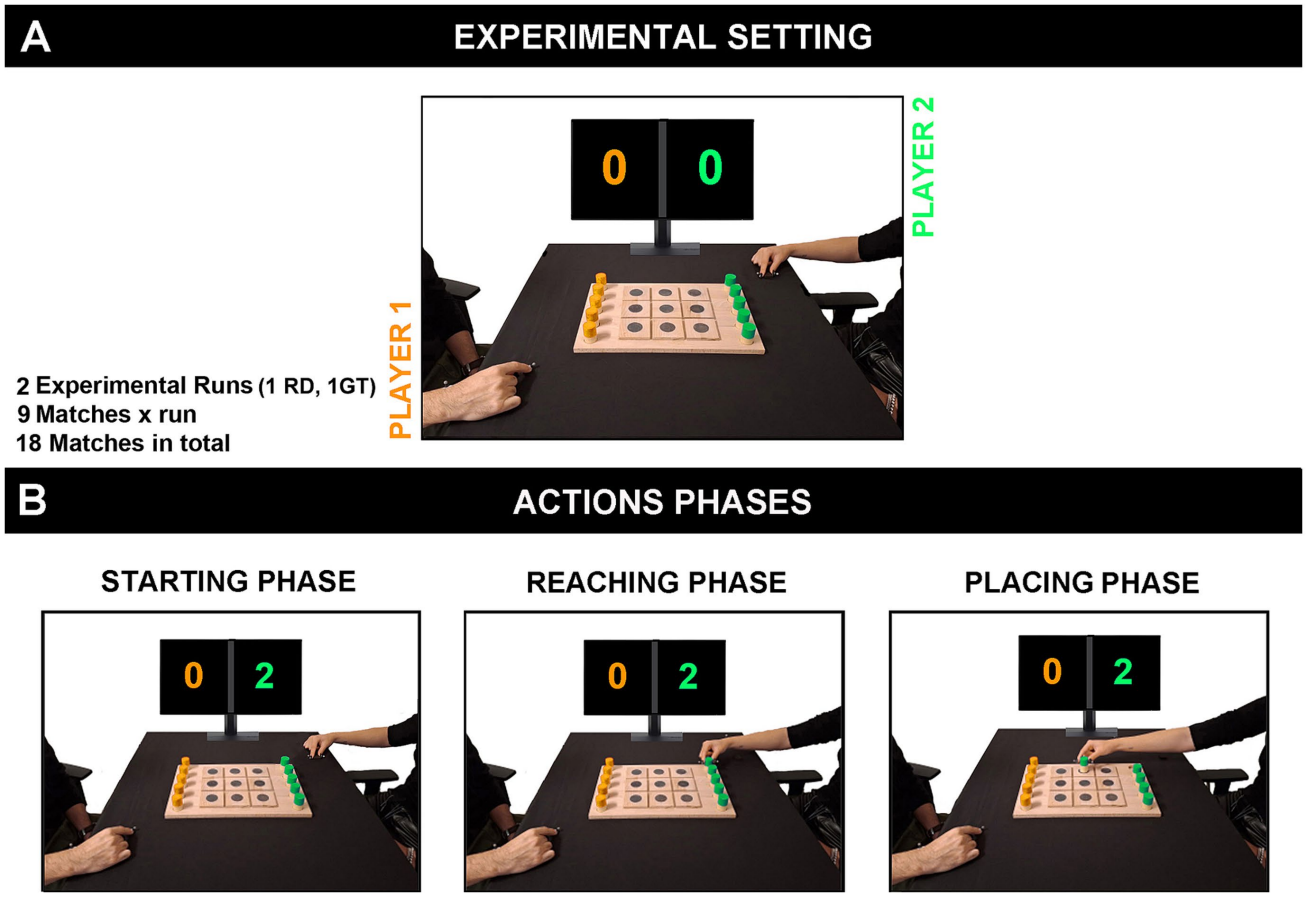
Experimental setting. The two players sat in front of a desk with a tic-tac-toe board placed at the center. One was the participant (right side) and the other was the inside player (left side) **(A)**. They first completed the baseline condition and then played 18 matches (in nine of them the inside player acted rudely, and in the other nine, gently). Each game action started from a fixed position (starting phase) and consisted of two distinct phases: the reaching phase, and the placing phase **(B)**. The kinematics of the two players were recorded using the OptiTrack system.

**Figure 2:**
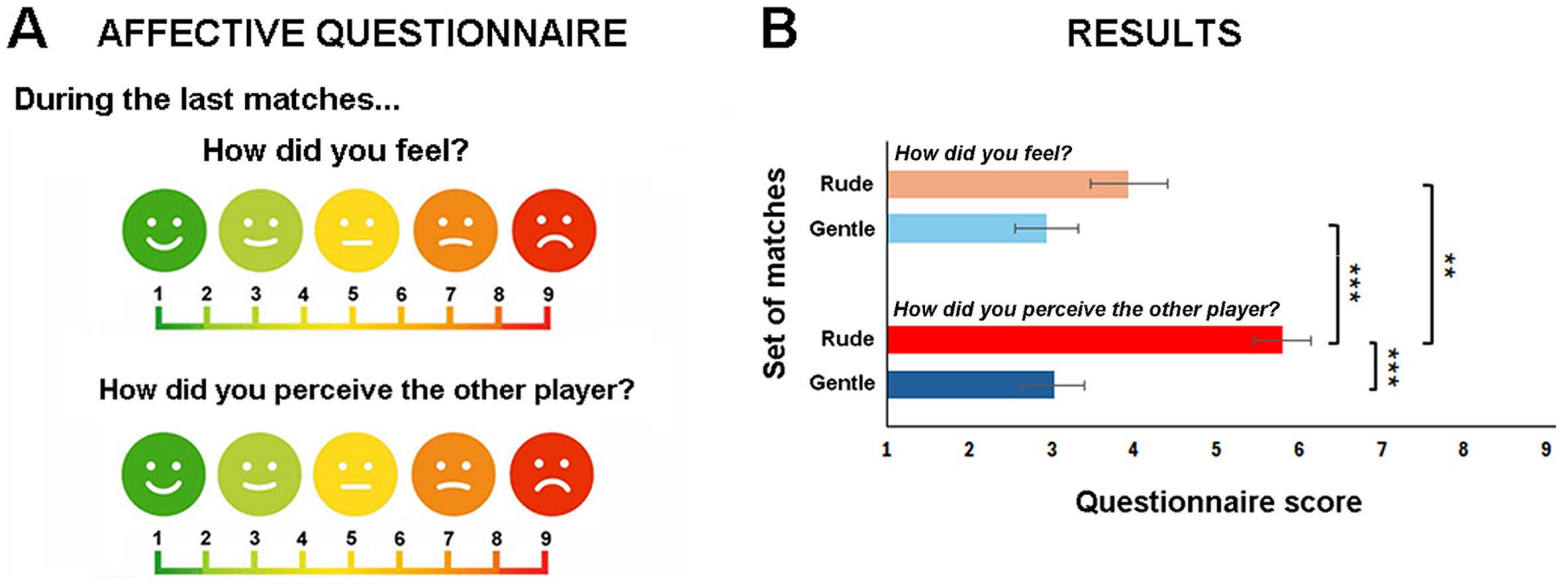
Affective questionnaire. Every three matches the players had to answer two questions: the first one was about their internal state “How did you feel?” and the second one was about the internal state of the other player “How did you perceive the other player?”. They had to respond by marking a cross on a scale from 1 “very positive” and 10 “very negative” **(A)**. Results. The graph represents the interaction effect between the vitality and the question. Significance threshold was set at *p* ≤ 0.05*, *p* ≤ 0.01**, *p* ≤ 0.001***. Vertical bars represent the standard errors **(B)**.

During the experimental session, the participant was seated on a chair in front of a Table (80 × 80 cm) with a tic-tac-toe board (28 × 42 cm) in the center. On the opposite side of the table sat another man player (insider), whose role was to manipulate the matches by modulating his VFs. The board game consisted of 9 squares arranged in a 3 × 3 grid, each marked with a central circle (3 cm), where players could place a maximum of five game pieces each (red pieces for one player and blue pieces for the other, to differentiate their moves) (Figure 1A). During the matches, the players took turns placing their game pieces on the squares. The first player to align three of their game pieces in a row, horizontally, vertically, or diagonally, won the match. If all 9 squares were filled and no one had placed three pieces in a row, the match ended in a draw. Each game action started from a fixed position (starting phase) and consisted of two phases: the reaching phase, during which participants moved their hand toward the game piece and grasped it; the placing phase, during which they placed the game piece into one of 9 possible squares (Figure 1B). The whole experiment consisted of two different runs each lasting 9 min. During each run, nine matches were executed. For the 50% of participants in the first run, the inside player displayed a gentle playing behavior, whereas from the second run he displayed a rude playing behavior. For the remaining 50% of participants, the order was reversed: the inside player exhibited rude playing behavior in the first run, followed by gentle playing behavior in matches of the second run. The outcomes of the games were balanced by the inside player, who used simple strategies to manipulate whether the participant won or lost, ensuring that this factor did not influence the participant’s internal state. In particular, the inside players were instructed to draw 50% of the matches, win 25%, and lose 25% across the two game runs, distributing the results equally. At the end of each match, the score was updated and displayed to both participants on a screen. Additionally, every three matches, participants reported their own internal state and their perception of the insider’s state by using a 10-point Likert scale (1 very positive − 10 very negative) (Figure 2A). Before starting the matches, participants completed two baseline conditions in which they placed the game pieces in each of the nine squares, allowing us to record their movements under basic and non-interactive conditions.

## Data recording

On a first laptop, E-Prime software was used to administrate the matches through a series of vocal commands. Specifically, the PC voice announced when each match began and ended, as well as which player started (player 1 or player 2). Kinematic data were recorded using an OptiTrack V120 Trio system connected to a second laptop running Motive v2.3 software. The two laptops were synchronized using an external sync box (Brain Products GmbH). To record participants’ hand movements, three passive markers were attached to the right hand: one on the wrist, which served as the primary source of kinematic data, and two on the thumbnails and index fingernails, which were used to quantify maximum hand aperture during the reach to grasp phase. The data were pre-processed using Motive v2.3 and subsequently analyzed in MATLAB (R2023b).

## Analysis and results

**Figure 3:**
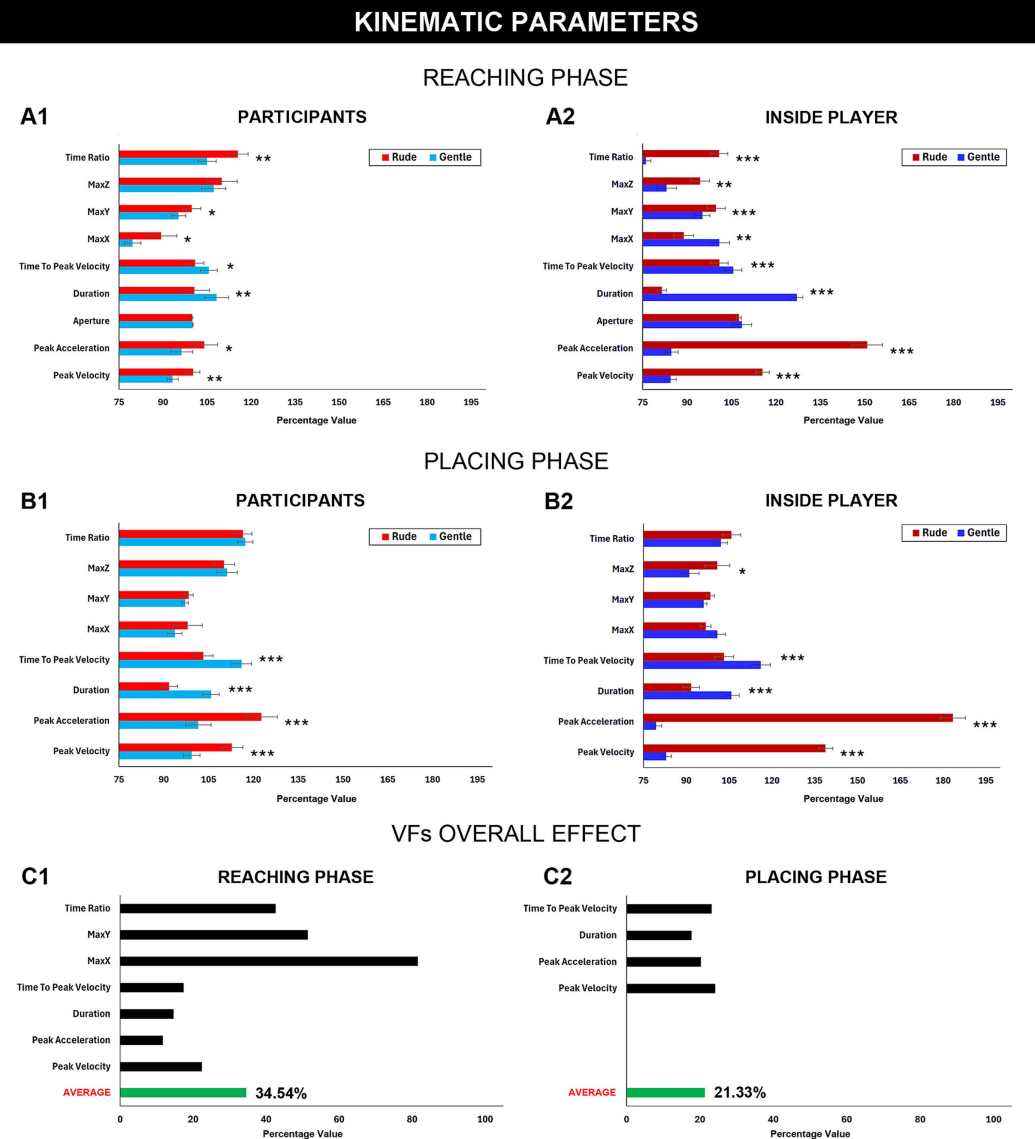
Kinematic parameters. The first two graphs showed the kinematic differences in the reaching phase between the rude and the gentle condition. At the left there is the mean representation of the kinematic parameters of the participant **(A1)**, at the right of the insider **(A2)**. In the second line the two graphs represent the kinematic differences in the placing phase between the rude and the gentle condition. At the left there is the mean representation of the kinematic parameters of the participant **(B1)**, at the right of the inside player **(B2)**. The last two graphs showed the VFs effect for the significant kinematic parameter for the reaching **(C1)** and for the placing **(C2)** phase. Significance threshold was set at *p* ≤ 0.05 *, *p* ≤ 0.01 **, *p* ≤ 0.001***. Vertical bars represent the standard errors.

**Figure 4:**
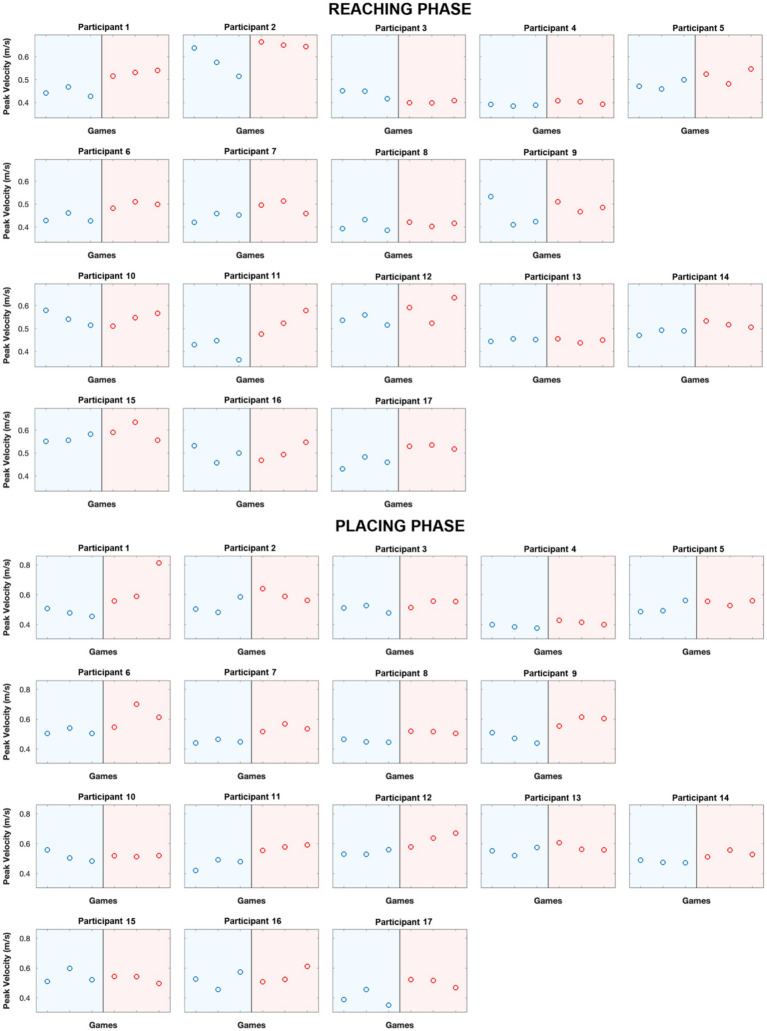
The graphs represent an example of how each participant’s peak velocity varied across the experiment, in response to the positive or negative attitude of the inside player. Each point in the graph represents the mean peak velocity within a triplet of games. The upper part illustrates the reaching phase, while the lower part represents the placing phase.

Based on the participants’ game actions, we examined potential kinematic differences in response to rude and gentle conditions manipulated by the inside player, in order to determine whether their movements were influenced by the insider’s behavior/mood. For each action, two distinct phases were analyzed: the reaching phase (*r*) and the placing phase (*p*). For both phases, the following kinematic parameters were computed: *Peak Velocity*, *Peak Acceleration, Duration*, *Aperture* (only for reaching phase), *Time to peak Velocity*, *Maximum Reaching* along the *X*, *Y*, and *Z*-axes (MaxX, MaxY, MaxZ), *Percentage of Acceleration* and *Deceleration Time ratio*. For each participant and for the above-mentioned kinematic parameters, we averaged the values of the actions performed across the matches. All parameters recorded during gentle matches were analyzed separately from those recorded during rude matches to allow for direct comparison. These average values were then normalized for the baseline conditions, in order to eliminate potential individual confounding effects and to better highlight the impact of VFs. These kinematic data were then analyzed using paired *t*-test, to assess possible differences in each parameter between the rude and the gentle matches (experimental conditions). This was calculated for both the participants and the inside player. Results are shown in Figure 3 and statistical values are described in Table 1. Figure 4 illustrates an example of how each participant’s peak velocity varied across the experiment, in response to the positive (run 1) or negative (run 2) attitude of the inside player. Notably, for participants 1 to 9, the game began with a gentle contest followed by a rude one, whereas for participants 10 to 17, the order was reversed.

**Table 1:** Results and statistical values of the main kinematic features analyzed during the experiment.

Participants					Reaching phase				Placing phase			
Kinematic parameters	Mean		SEM		*p*-value adjusted	Effect size	Mean		SEM		*p*-value adjusted	Effect size
	Rude	Gentle	Rude	Gentle			Rude	Gentle	Rude	Gentle		
Peak velocity	100.2	93.3	2.3	1.9	0.01	−0.92	112.8	99.4	3.6	2.8	0.0001	−1.35
Peak acceleration	104.1	96.3	4.5	3.8	0.04	−0.58	122.7	101.6	5.3	4.3	0.0001	−1.28
Duration	100.7	108.2	5.1	4.1	0.01	0.81	91.7	105.8	2.9	2.7	0.0001	1.67
Time to peak vel	100.9	105.6	2.9	2.9	0.04	0.57	103.2	116.1	3.3	3.3	0.0003	1.17
MaxX	89.4	79.6	5.3	2.8	0.04	−0.61	98.0	93.7	4.9	2.4	0.33	−0.31
MaxY	99.8	95.2	3.1	2.5	0.05	−0.53	98.4	97.2	1.5	1.1	0.43	−0.24
MaxZ	110.1	107.2	5.2	4.1	0.62	−0.12	110.2	111.2	3.6	3.4	0.81	0.07
Time ratio	115.5	105.0	3.5	3.1	0.01	−0.84	116.5	117.3	2.9	2.6	0.81	0.05
Aperture	99.9	100.1	0.2	0.1	0.43	0.21	–	–	–	–	–	–

Inside player					Reaching phase				Placing phase			
Kinematic parameters	Mean		SEM		*p*-value adjusted	Effect size	Mean		SEM		*p*-value adjusted	Effect size
	Rude	Gentle	Rude	Gentle			Rude	Gentle	Rude	Gentle		
Peak velocity	115.5	84.4	2.3	1.9	0.0001	−5.10	138.8	82.9	2.6	1.8	0.0001	−4.88
Peak acceleration	150.8	84.7	5.2	2.3	0.0001	−3.39	183.5	79.4	4.4	1.9	0.0001	−5.08
Duration	81.5	127.1	1.5	2.0	0.0001	4.94	91.7	105.8	2.9	2.7	0.0001	7.74
Time to peak vel	100.9	105.6	2.9	2.9	0.0001	2.89	103.2	116.1	3.3	3.3	0.0001	6.55
MaxX	88.9	100.9	3.3	3.5	0.001	0.93	96.8	100.9	1.9	2.9	0.17	0.36
MaxY	99.8	95.2	3.1	2.5	0.001	−0.98	98.5	96.1	1.4	1.1	0.14	−0.41
MaxZ	94.4	83.1	3.2	3.4	0.003	−0.84	100.8	91.1	4.4	3.3	0.02	−0.66
Time ratio	100.8	76.1	2.9	1.6	0.0001	−1.82	105.8	102.1	3.3	2.3	0.29	−0.29
Aperture	107.5	108.5	0.7	3.3	0.77	0.07	–	–	–	–	–	–

Significance threshold was set at *p* ≤ 0.05*, *p* ≤ 0.01**, *p* ≤ 0.001*** (false discovery rate correction). The effect size is expressed in terms of Cohen’s *d*.

### Estimation of the VFs effect

To evaluate how the kinematic parameters varied between rude and gentle conditions, we calculated a Delta value (*Δ*VFs) as follows:


$$\Delta VFs=\left|Rudeparameter-Gentleparameter\right|*100$$

This value quantified how much the participant and the inside player modulated their actions during the gentle and rude matches. Notably, *Δ*VFs was calculated only for significant kinematic parameters. Once this value was obtained, the participants’ ΔVFs were compared with those of the inside player. This computation allowed us to assess the *general effect of VFs* exerted by the inside player through his modulation of VFs on the participant’s game actions. This effect was computed separately for each kinematic parameter as follows:


$$VFs\;effect=\frac{\Delta VFs\;participant}{\Delta VFs\;insider}*100$$

Figure 4 indicates the *VFs effect* for both the reaching (*Peak Velocity* = 22.43%, *Peak Acceleration* = 11.73%, *Duration* = 14.65%, *Time to Peak Velocity* = 17.42%, MaxX = 81.56%, MaxY = 51.43%, *Time Ratio* = 42.58%) (Figure 3C1) and the placing phases (*Peak Velocity* = 24.13%, *Peak Acceleration* = 20.26%, *Duration* = 17.73%, *Time to Peak Velocity* = 23.18%) (Figure 3C2). Considering all the parameters, we finally calculated the average of the VFs effect for both the actions phases showing that this effect was higher for the reaching phase (34.54%) than for the placing phase (21.33%) (Figure 3C2).

### Post-game debriefing questionnaire

After every three matches, both players were required to report their own affective state as well as their perception of the other player’s affect. Specifically, participants answered two questions referring to the previous three matches, each rated on a 10-point scale ranging from *very positive* (Aoki et al., 2025) to *very negative* (Di Cesare et al., 2014): “How did you feel?” and “How did you perceive the other player?” (see Figure 2A). To examine possible differences between gentle and rude matches for the two questions, we performed a General Linear Model (GLM) analysis. The analysis included two factors: VITALITY (Rude or Gentle), and the QUESTION (*How did you feel?’* and ‘How did you perceive the other player?’). The results revealed a main effect of VITALITY (*F* = 57.7, *p* < 0.001), indicating higher scores for the rude condition (mean = 4.7, SEM = ±0.44) compared to the gentle one (mean = 2.8, SEM = ±0.35). Additionally, a significant QUESTION effect was observed (*F* = 14.5, *p* = 0.001), with significantly higher score for the question ‘How did you perceive the other player?’ (mean = 4.2, SEM = ±0.4) than for the other one ‘How did you feel?*’* (mean = 3.3, SEM = ±0.4). Finally, and most importantly, there was a significant interaction effect between VITALITY and QUESTION (*F* = 26.2, *p* < 0.001). *Post hoc* analysis (Bonferroni correction) revealed a significant difference between the rude and the gentle conditions for the question *‘How did you perceive the other player?’* (*p* < 0.001; rude mean = 5.5, SEM = ±0.3; gentle mean = 2.9, SEM = ±0.3). Additionally, two significant differences between the two questions were found but only for the rude VF (*p* < 0.01; rude ‘How did you perceive…’ mean = 5.5, SEM = ±0.4; rude ‘*How did you feel?’* mean = 3.8, SEM = ±0.3) and between the gentle condition for the first question and the rude one for the second question (*p* < 0.001; gentle ‘*How did you feel?’* mean = 2.8, SEM = ±0.3; rude *‘How did you perceive…’* mean = 5.5, SEM = ±0.3) (Figure 2B).

### Correlation analysis

**Figure 5:**
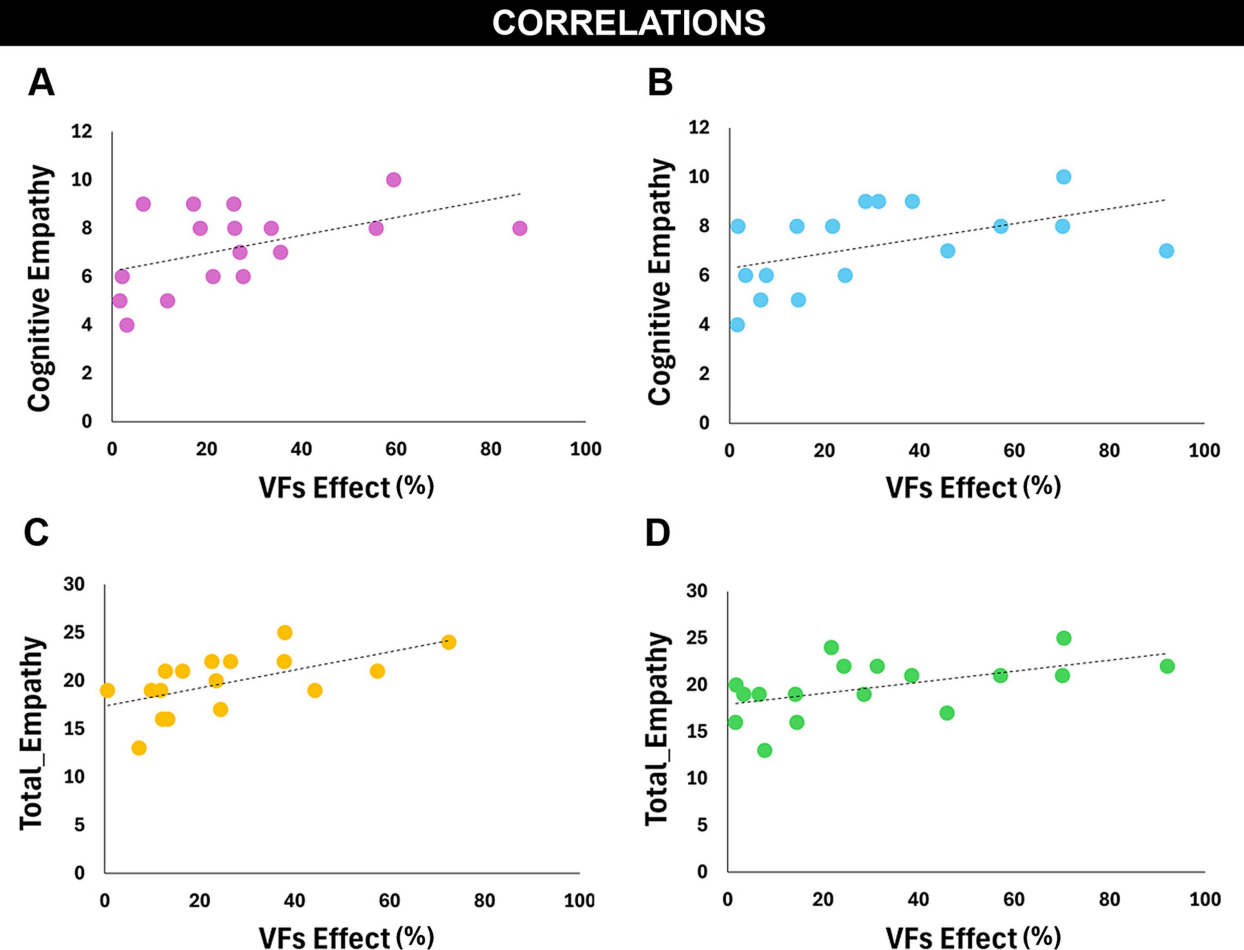
The EQ scores were correlated with the VFs effect of different kinematic parameters. Particularly, a significant correlation was found between the subscale “cognitive empathy” and the VFs effect for the parameter peak velocity **(A)** and time to peak velocity in the reaching phase **(B)**, and between the “Total empathy score” and the VFs effect for the parameter peak velocity in the placing phase **(C)** and time to peak velocity in the reaching phase **(D)**.

To explore possible relationship between the motor contagion effect across conditions (rude vs. gentle) and participants’ empathy levels, we correlated the VFs effect for all kinematic parameters with EQ total scores and its subscales (cognitive empathy, emotional reactivity, and social skills). The results showed that Cognitive Empathy (CE) scores correlate in a positive and significant way with the VFs effect for the *Peak Velocity* during the reaching phase (*r* = 0.49, *p* < 0.05) (Figure 5A) and with the VFs effect for the *Time to Peak Velocity* during the reaching phase (*r* = 0.49, *p* < 0.5) (Figure 5B). Additionally, we found that the Empathy Quotient total scores correlate in a positive and significant way with the VFs effect for the peak velocity during the placing phase (*r* = 0.58, *p* = 0.01) (Figure 5C) and with the VFs effect for the Time to Peak Velocity during the reaching phase (*r* = 0.53, *p* < 0.05) (Figure 5D). Finally, to rule out the possibility that the VFs effect observed in participants was merely driven by imitation of the actions performed by the inside player, we correlated the magnitude of VF modulation in the inside player with that observed in the participants. The results revealed no significant correlations for the main kinematic features, namely Peak Velocity (reaching: *r* = 0.03, *p* > 0.05; placing: *r* = 0.18, *p* > 0.05), Peak Acceleration (reaching: *r* = 0.36, *p* > 0.05; placing: *r* = 0.46, *p* > 0.05), and Movement Duration (reaching: *r* = 0.22, *p* > 0.05; placing: *r* = 0.09, *p* > 0.05).

## Discussion

Previous studies suggest that during pseudo-social interactions, the VFs expressed by human (Di Cesare et al., 2017) or humanoid agents (Lombardi et al., 2024; Vannucci et al., 2024) influence both the perception and the motor behavior of participants. However, these studies examined this topic exclusively in non-real-world contexts using highly controlled paradigms. Consequently, how VFs affect human actions during real social interactions, where two individuals interact and dynamically influence each other, remains largely unexplored. To fill this gap, the present study aimed to investigate, for the first time, how VFs affect the kinematic features of participant actions during a real and reciprocal interaction, providing evidence of their impact in social contexts. To achieve this aim, we conducted a study in which participants played tic-tac-toe in pairs, with one player acting as an insider instructed to modulate the game actions in either a rude or gentle manner.

The kinematic analysis showed that participants’ actions were significantly modulated, across multiple parameters during both the reaching and placing phases including *Peak Velocity, Peak Acceleration, Time to Peak Velocity, and Action Duration,* by VFs (rude or gentle) expressed by the inside player. Moreover, to assess differences in kinematic parameters between the rude and gentle conditions, we calculated the *effect of VFs* exerted by the inside player through his modulation of gentle and rude actions on the participant’s game actions. The results of this analysis indicated that the inside player influenced the participant’s game actions by 34.5% during the *reaching phase* and by 21.3% during the *placing phase*. The difference in the effect of VFs observed between the two phases of the action can be explained on the basis that they constitute two distinct motor acts. The first, more spontaneous and associated with the initial phase of the action (reaching phase), is therefore more susceptible to VFs contagion. In contrast, the second is related to the goal component of the action (placing phase), more constrained and consequently less susceptible to influence.

These findings suggest that the modulation exerted by the inside player affected the participant’s motor behavior, reflecting an affective kinematic modulation.

An interesting question is whether the observed motor contagion resulted from a mere imitation of specific kinematic parameters (such as velocity and acceleration) of the actions performed by the inside player. However, this interpretation is not supported by our findings, which show that the kinematic features modulated by participants during the action games differed from those voluntarily expressed by the inside player. Notably, during the task, participants generally reported noticing changes in the inside player’s behavior and tended to describe their own affective state as neutral or calm, even in games that involved more aggressive game interactions, during which their movements showed tendencies toward higher speed and acceleration. This suggests that the VFs effect observed in participants may operate in an unconscious manner.

During social exchange, a fundamental point is to be synchronized and tuned with the others in order to reciprocally interact with others. Being synchronized with another person means having a deep affective and intuitive connection, in which actions and forms are shared without the need of words. Our findings clearly demonstrate that, within a game context, the attitude of others influences the receiver’s motor behavior, leading to the emergence of more positive or negative game dyads. Moreover, our data indicate that the ability to be synchronized with or influenced by the other is significantly related with individual empathic abilities, showing that participants with a higher empathic quotient are the ones most strongly affected by the inside player. Most importantly, these findings are in line with previous kinematic studies showing the same modulation effect is also present during pseudo-social interactions between humans (Di Cesare et al., 2017; Lombardi et al., 2021; Lombardi et al., 2024) and real interaction between humans and humanoids (Vannucci et al., 2024; Aoki et al., 2025). In particular, Vannucci et al. (2024) asked to a group of participants to pay attention to a taking request performed gently or rudely by the iCub humanoid robot and subsequently to reach and place the requested object on a specific target. Results clearly showed a modulation effect produced by the perception of different VFs (gentle/rude) generated by the iCub robot on kinematic parameters of the human participants’ response. However, comparing previous research with our findings, it is important to highlight the magnitude of the VFs effect. While previous pseudo-social human-human and human-robot interactions produced a modulation of participants’ actions of approximately 10%, real interactions resulted in a substantially larger effect, with an average modulation of about 27%, considering both the reaching and placing phases.

Considering social interactions and the potential modulation effect between two interactans, what is this contagion effect due to? As described by Di Cesare et al. (2014) the observation of gentle and rude actions and the execution of the same actions performed with the same VFs, produced the activation not only of the parieto-frontal network, but also the dorso-central insula (DCI) (Di Cesare et al., 2015), and the middle cingulate cortex (MCC) (Di Cesare et al., 2021). These findings suggest that while the parieto-frontal circuit processes the goal of an action, the DCI and the MCC are involved in processing its forms. Interestingly, using Dynamic Causal Modeling, the same authors assessed the relation between the insula and the parieto-frontal network, showing that, during action *observation*, two streams arose from the superior temporal sulcus (pSTS): one towards inferior parietal lobe (IPL) (encoding action goal) and one towards DCI (encoding action VFs). After receiving the VFs information, DCI modulate the premotor cortex (PM). It is plausible to hypothesize that DCI encodes the affective information of observed actions and send it to PM. In this process, the affective aspects of an action are transformed into motor features, such as the kinematic parameters that characterize distinct motor acts. Conversely, when an agent responds to that specific observed action, the same kinematic parameters may be recruited to execute the action in a similar manner, thereby enabling affective tuning between the agent and the receiver.

In conclusion, our findings indicate that, beyond action goal, VFs constitute a crucial source of information for understanding an agent’s affective state representing a key mechanism for interpersonal communication. Most importantly, we demonstrated that positive and negative game actions, associated with gentle and rude VFs, can modulate action execution by influencing individuals’ motor behavior. These results shed new light on action execution during real interpersonal interactions opening new perspectives on pathologies characterized by difficulties in recognizing and promptly responding to different action requests (gentle or rude). Indeed, impairments in affective recognition are among the most common cognitive deficits and have been extensively investigated in several clinical populations, including Autism Spectrum Disorder (Di Cesare et al., 2024; Cuccio et al., 2025) and ADHD (Chen et al., 2026). From this perspective, the present study provides a promising framework for future research aimed at deepening the understanding of affective communication in clinical populations, offering also a valuable insight for social communication research and for the fields of human–robot interaction.

## Data Availability

The original contributions presented in the study are included in the article/supplementary material, further inquiries can be directed to the corresponding author/s.
